# Transcriptomic analysis of cell envelope inhibition by prodigiosin in methicillin-resistant *Staphylococcus aureus*

**DOI:** 10.3389/fmicb.2024.1333526

**Published:** 2024-01-22

**Authors:** Xiaoxia Liu, Zonglin Wang, Zhongyu You, Wei Wang, Yujie Wang, Wenjing Wu, Yongjia Peng, Suping Zhang, Yinan Yun, Jin Zhang

**Affiliations:** ^1^College of Biological, Chemical Sciences and Engineering, Jiaxing University, Jiaxing, China; ^2^College of Geography and Environmental Sciences, Zhejiang Normal University, Jinhua, China; ^3^Clinical Laboratory of First Hospital of Jiaxing, Jiaxing, China; ^4^College of Advanced Materials Engineering, Jiaxing Nanhu University, Jiaxing, China

**Keywords:** prodigiosin, methicillin-resistant *Staphylococcus aureus*, biofilm, transcriptomic analysis, molecular mechanism

## Abstract

Methicillin-resistant *Staphylococcus aureus* (MRSA) is a leading threat to public health as it is resistant to most currently available antibiotics. Prodigiosin is a secondary metabolite of microorganisms with broad-spectrum antibacterial activity. This study identified a significant antibacterial effect of prodigiosin against MRSA with a minimum inhibitory concentration as low as 2.5 mg/L. The results of scanning electron microscopy, crystal violet staining, and confocal laser scanning microscopy indicated that prodigiosin inhibited biofilm formation in *S. aureus* USA300, while also destroying the structure of the cell wall and cell membrane, which was confirmed by transmission electron microscopy. At a prodigiosin concentration of 1.25 mg/L, biofilm formation was inhibited by 76.24%, while 2.5 mg/L prodigiosin significantly reduced the vitality of MRSA cells in the biofilm. Furthermore, the transcriptomic results obtained at 1/8 MIC of prodigiosin indicated that 235and 387 genes of *S. aureus* USA300 were significantly up- and downregulated, respectively. The downregulated genes were related to two-component systems, including the transcriptional regulator LytS, quorum sensing histidine kinases SrrB, NreA and NreB, peptidoglycan biosynthesis enzymes (MurQ and GlmU), iron-sulfur cluster repair protein ScdA, microbial surface components recognizing adaptive matrix molecules, as well as the key arginine synthesis enzymes ArcC and ArgF. The upregulated genes were mainly related to cell wall biosynthesis, as well as two-component systems including vancomycin resistance-associated regulator, lipoteichoic acid biosynthesis related proteins DltD and DltB, as well as the 9 capsular polysaccharide biosynthesis proteins. This study elucidated the molecular mechanisms through which prodigiosin affects the cell envelope of MRSA from the perspectives of cell wall synthesis, cell membrane and biofilm formation, providing new potential targets for the development of antimicrobials for the treatment of MRSA.

## 1 Introduction

The abuse of antibiotics has accelerated the emergence of drug-resistant bacteria, among which MRSA is a serious threat to public health (Chalmers and Wylam, 2020). MRSA has gained attention not only because of its strong pathogenicity (Turner et al., 2019; Nandhini et al., 2022), but also because of its resistance to currently available antibiotics, which has brought an enormous burden to medical institutions. Therefore, the World Health Organization prioritized MRSA as a Class II pathogen (Cadelis et al., 2021). Approximately 80% of chronic and recurrent infections in humans are caused by bacterial biofilms (Lindsay and Holden, 2004), which can also be formed by MRSA on the surface of the skin, nasopharyngeal mucosa and gastrointestinal tract. In fact, MRSA is one of the most common causative agents of biofilm infections (Turner et al., 2019).

Cell envelope includes biofilm, cell membrane, and cell wall. Biofilms are composed of multiple layers of extracellular polymeric substances, including proteins, polysaccharides, and extracellular DNA, which protect the bacteria enclosed inside to avoid the host immune system, as well as environmental stress factors such as antibiotics and disinfectants (Paharik and Horswill, 2016; Flemming et al., 2023). It was observed that bacterial cells inside biofilms are resistant to antibiotic concentrations up to 1,000× greater than those required to kill planktonic bacteria (Mottola et al., 2016). Therefore, MRSA biofilm infections are one of the main concerns in the global public health sector. Currently, there are no effective drugs targeting MRSA biofilms in clinical practice (Jolivet-Gougeon and Bonnaure-Mallet, 2014; Nguena-Dongue et al., 2023). Therefore, the development of new drugs to treat MRSA infection is increasingly urgent.

Prodigiosin is a secondary metabolite of many microorganisms, including *Serratia marcescens*, *Serratia nematodiphila*, *Zooshikella* sp., *Serratia plymuthica*, etc., characterized by a relatively simple structure of three pyrrole rings and a methoxy group (Darshan and Manonmani, 2016; Woodhams et al., 2018; Ramesh et al., 2020; Amorim et al., 2022). In the past decade, various biological effects of prodigiosin were revealed, including immunosuppressive, anticancer, antimalarial and fungicidal activities (Han et al., 2005; Wang et al., 2016; Li et al., 2018; Han et al., 2021; Nguyen et al., 2022). Moreover, prodigiosin also exhibits broad-spectrum antibacterial activity against both Gram-positive and -negative bacteria, such as *Bacillus subtilis*, *Escherichia coli*, and *Staphylococcus aureus* (Suryawanshi et al., 2014; Woodhams et al., 2018; Herráez et al., 2019). The mechanisms underlying the antibacterial activity of prodigiosin include the disruption of the cell membrane and inhibition of biofilm formation (Danevčič et al., 2016b; Kimyon et al., 2016; Suryawanshi et al., 2017; Hage-Hülsmann et al., 2018).

However, the molecular mechanism through which prodigiosin inhibits the growth MRSA growth remains unclear to date. Thus, the aim of this study was to elucidate the molecular mechanism of the antibacterial effect of prodigiosin against the growth and especially envelope formation of MRSA. The findings of this study will hopefully contribute to the design of better antibacterial agents targeting multidrug resistant bacteria in the future.

## 2 Materials and methods

### 2.1 Bacterial strains, culture conditions, and prodigiosin purification

Methicillin-sensitive *S. aureus* (MSSA) ATCC 25923 and MRSA USA300 were purchased from the American Type Culture Collection (ATCC). The strain *S. marcescens* jx-1 was stored in our laboratory. Pure prodigiosin (HPLC >98%) was obtained using a previously published protocol (Sun et al., 2015).

### 2.2 Antibiotic resistance spectrum of *Staphylococcus aureus* isolates

The *S. aureus* isolates from patients at the Jiaxing First Hospital and identified and analyzed according to a previously published protocol (Wang B. et al., 2022; Wang Y. J. et al., 2022). The antibiotic resistance spectrum of MRSA isolates was confirmed using the method described by the National Clinical and Laboratory Standards Institute (Cusack et al., 2019).

### 2.3 Measurement of bacterial growth curves after treatment with prodigiosin

Samples comprising 100 μL of different concentrations of prodigiosin mixed with Mueller-Hinton (MH) broth in 96-well flat-bottom microplates, after which 100 μL of a bacterial suspension (∼10^6^ CFU/mL) was added to the same wells and incubated at 37°C for 24 h. The final concentration prodigiosin is 0, 0.16, 0.31, 0.63, 1.25 and 2.5 mg/L. The optical density at 620 nm was measured every 2 h.

### 2.4 Determination of the minimum inhibitory concentration

The minimum inhibitory concentration (MIC) of prodigiosin was determined using a two-fold dilution technique according to the CLSI with MH broth in 96-well microplates. Prodigiosin was serially diluted to 20, 10, 5, 2.5 and 1.25 mg/L. Then, 200 μL MRSA suspensions (∼10^6^ CFU/mL) were used to inoculate MH broth, placed into the wells, and incubated for 16 h with different concentrations (1.25, 2.5, 5, 10 or 20 mg/L) of prodigiosin, and only MH broth with different concentrations of prodigiosin as control. The OD620 absorbance values were measured at 16 h. The MIC value for antibacterial activity was defined as the lowest concentration that inhibited cell growth after 16 h culture.

### 2.5 Detection of biofilm formation using the crystal-violet assay

To evaluate the effect of the different concentrations and addition times of prodigiosin on MRSA biofilm formation, the biofilm assay was performed in 96-well flat-bottom plates, according to a previously published protocol (Lu et al., 2021). The 200 μL bacterial cells (∼10^6^ CFU/mL) were cultured in tryptic soy broth (TSB) containing 1% glucose (TSBG) and treated with different concentration (0.16, 0.31, 0.63, 1.25 or 2.5 mg/L) of prodigiosin for 24 h at 37°C without shaking. Addition time effects of prodigiosin on the biofilm biomass of MRSA USA300 were investigated under the same culture conditions. Prodigiosin was added at concentrations of 2.5 mg/mL or 5 mg/mL every 2 h from the start of cultivation (0 h) to the stationary phase (8 h), and bacteria were cultured up to 24 h. Edge effects were avoided by adding 200 μL TSBG to the 96-well plates. The plates obtained above were centrifuged, the supernatant was discarded, and the cell pellet was washed with water three times. Then, the biofilm in the plates was stained with 0.1% crystal violet (m/V) for 20 min. The excess crystal violet was washed with water three times, and crystal violet combined with biofilm was solubilized in 95% ethanol (Sivasamy et al., 2021). The absorbance of the ethanol solution at 570 nm was measured using a Spark^®^ microplate reader (Tecan, Switzerland).

### 2.6 Scanning electron microscopy of the cell envelope

MRSA USA300 was cultured in 12-well polystyrene plates in TSBG at 37°C for 24 h with 2.5 mg/L prodigiosin. An otherwise identical culture without prodigiosin was included as a control. Then, the plates were centrifuged, the supernatant was removed, the biofilms were washed with phosphate-buffered saline (PBS, pH7.4), and fixed with 2.5% glutaraldehyde at 4°C for 1 h. Then, the biofilms were observed on an SU-8010 SEM (Hitachi, Tokyo, Japan).

### 2.7 Confocal laser scanning microscopy of biofilms

MRSA was cultured using TSBG in 12-well polystyrene plates with the 20 mm-diameter glass coverslips (Guan et al., 2020) without shaking at 37°C for 24 h with 2.5 mg/L prodigiosin, which was added at different cultivation times (0 h, 4 h and 10 h). Then fermentation broth was removed, and the glass coverslips were washed three times with PBS, and further processed as described before (Sathiyamoorthi et al., 2021). The biofilms were stained with 5 μM final concentration carboxy-fluorescein diacetate succinimidyl ester (CFSE) for 20 min at 37°C, then counter-stained with propidium iodide (PI) for 20 min and washed three times with PBS. The biofilm was visualized using excitation with an Ar 488 nm light source (emission wavelengths 200–550 nm). The cells were visualized by CLSM using a 20 × objective. Methanol was used as the control. Color confocal images were visualized using Olympus FluoView Fv31s-SW. For each experiment, at least 10 random positions in three independent cultures were chosen for CLSM analysis using Olympus cellSens dimension software.

### 2.8 Effect of prodigiosin on the viability of cells in the biofilm

An aliquot comprising 100 μL TSBG and 100 μL of a bacterial suspension (∼10^6^ CFU/mL) to the 96-well plate and incubated until the biofilm establishment. After 24 h, the medium was gently aspirated from the wells and the wells were rinsed three times with PBS. The two-fold dilutions of prodigiosin (from 10 mg/L to 2.5 mg/L) in 200 μL of TSBG were then added to the wells. No prodigiosin was added to the positive biofilm control wells. The 96-well plate was incubated at 37°C for 24 h, after which the medium was removed, and the wells were cleaned according to the method mentioned above. Biofilm viability was assessed using the CFU counting method adapted from Pettit et al. (2005). The detection limit was 100 CFU/mL.

### 2.9 Transmission electron microscopy of cell morphology

MRSA was cultured in 5 mL of TSBG at 37°C for 12 h with 1/8 MIC prodigiosin in 50 mL centrifuge tubes. An otherwise identical culture without prodigiosin was included as a control. After centrifugation at 8000 g, the supernatant was removed, and the collected cells were fixed with 2.5% glutaraldehyde at 4°C overnight. Then, the fixed cells were observed on a H-7650 TEM (Hitachi, Tokyo, Japan).

### 2.10 Preparation of MRSA USA300 cells for RNA-Seq

Shake flasks (250 mL) with 50 mL of TSBG were inoculated with a fresh colony of MRSA USA300 and incubated at 37°C with shaking at 250 rpm overnight. The cell suspension was adjusted to 10^6^ CFU/mL and 3 samples of the USA300_EG group (USA300_EG1, USA300_EG2, USA300_EG3) were treated with 1/8 MIC (0.31 mg/L) of prodigiosin for 5 h. The control group (USA300_CK, 3 samples) was incubated without prodigiosin under the same conditions for 5 h. Then, the cells were harvested by centrifugation at 8,000 g for 10 min, shock frozen in liquid nitrogen and stored at −80°C until further processing.

### 2.11 RNA extraction, Illumina sequencing and data analysis

Total RNA was extracted from 6 bacterial samples using a MicroRNAeasy Kit (Qiagen; 217004) following the manufacturer’s instructions. RNA concentrations were measured using a Qubit^®^ RNA assay kit in a Qubit^®^ 4.0 fluorometer, and RNA integrity was assessed through electrophoresis on a 1.0% (w/v) agarose gel. The rRNA was removed using a Ribo-off rRNA Depletion Kit V2 for Bacteria (Vazyme, China). The cDNA library was constructed as described before (Li et al., 2022).

### 2.12 Transcriptomic data analysis

The raw data were processed by discarding low-quality reads, including more than 30% bases with QA (quality analysis) ≤15. All subsequent analyses were based on the resulting clean reads. Gene Bowtie2 and RSEM were used to calculate the fragments per kilobase million (FPKM) and identify significantly expressed genes, respectively (Mortazavi et al., 2008). Differentially expressed genes were identified using the DESeq R package based on the criteria |log2(foldchange)| ≥1 and adjusted false discovery rate *p* < 0.05. Then, the DEGs were subjected to KEGG pathway and GO functional enrichment analysis with p < 0.05 as the threshold.

### 2.13 Quantitative real-time PCR

A total of 8 differentially expressed genes (DEGs) related to the cell envelope biosynthesis were selected to validate the results of RNA-sequencing by qRT-PCR analysis. Total RNA was extracted from the cells of MRSA USA300 cultured with 1/8 MIC (0.31 mg/L) prodigiosin in TSBG for 5 h using a Bacterial RNA extraction kit (Sangon Biotech, China) according to the manufacturer’s protocol. The extracted RNA was reverse transcribed into cDNA used random primers. Then, qRT-PCR was performed using specific primer pairs (Supplementary Table S1) and the 2× MagicSYBR mixture (CWBIO, Jiangsu, China). Each sample was analyzed in technical triplicates. The cDNA values were normalized to the 16S rRNA as the internal standard (Li et al., 2022).

### 2.14 Statistical analysis

All experiments were repeated three times, and the data were presented as the means ± SEM. When only two groups were compared, differences between groups were analyzed using student’s two-tailed *t*-test. When more than two groups were compared, differences between groups were analyzed using single-factor analysis of variance (one-way ANOVA). *p*-values <0.05 and <0.01 were, respectively, considered statistically significant and highly significant. All graphical evaluations were performed using GraphPad Prism 8.0.

## 3 Results

### 3.1 Identification and characterization of isolated *Staphylococcus aureus* strains

A total of 55 *S. aureus* strains were isolated from patients and identified using MALDI-TOF MS with a probability of 99% (Supplementary Table S2). The antibiotic resistance spectra of all isolates are listed in Supplementary Table S3. We found that there were 14 MSSA isolates, and 41 MRSA isolates according to oxacillin resistance. A total of 6 MRSA strains, respectively isolated from pus (*n* = 4), wound surface (*n* = 1), and blood (*n* = 1), were selected for further research.

### 3.2 Prodigiosin inhibited the cell growth of MRSA strains

**Figure 1:**
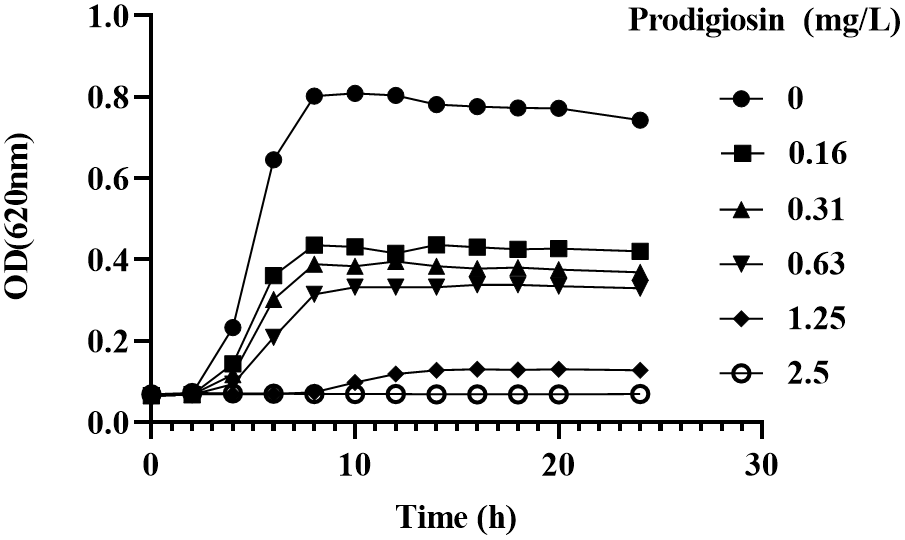
Dosage effects of prodigiosin on MRSA USA300 growth. Bacteria were cultured in MH medium with prodigiosin (0, 0.16, 0.31, 0.63, 1.25 or 2.5 mg/L) in a 96-well plate at 37°C for 24 h. OD620 values were measured every 2 h. Data were expressed as mean ± SEM (*n* = 3).

**Figure 2:**
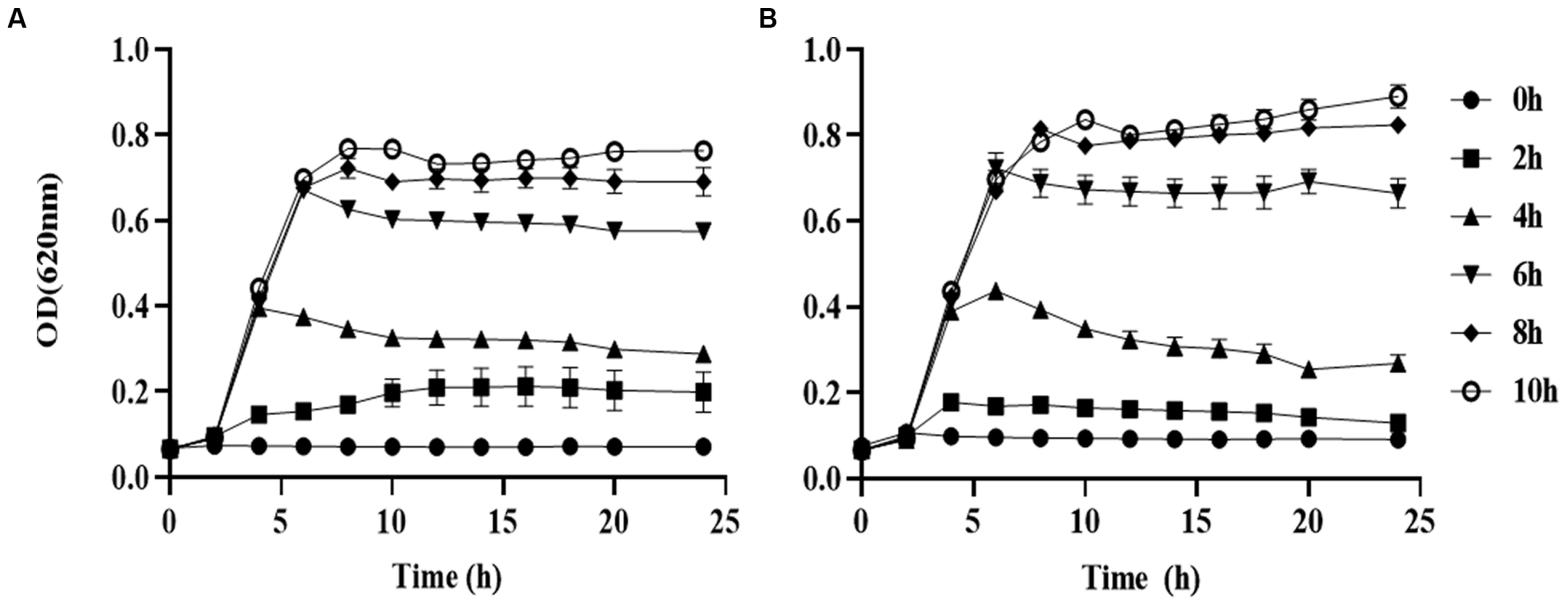
Addition time effects of prodigiosin on MRSA USA300 growth. Bacteria were cocultured with MH medium in a 96-well plate at 37°C. Prodigiosin was added at concentrations of 2.5 mg/mL **(A)** and 5 mg/mL **(B)** every 2 h from the start of cultivation (0 h) to 10 h. OD620 values were measured every 2 h up to 24 h. The sample treated without prodigiosin adding served as the control. Data were expressed as mean ± SEM (*n* = 3).

We used different concentrations of prodigiosin from 1.25 to 20 mg/L to treat seven *S. aureus* strains. The MIC ranged from 2.5 to 5 mg/L for all tested strains (Table 1). The effects of different concentrations and addition times on the growth were investigated (Figure 1). The results showed that early prodigiosin addition (before 6 h, late-logarithmic phase), had a greater impact on bacterial growth. However, when 2.5 mg/L (1 × MIC) and 5 mg/L (2 × MIC) of prodigiosin were added at 8 h, corresponding to the stationary phase of bacterial growth, there was no significant difference compared to the control (Figure 2).

**Table 1:** MIC values of prodigiosin against *S. aureus* strains.

Number	21,612,804	21,607,345	21,612,346	21,610,437	21,610,437	21,609,490	MRSA USA300
MIC (mg/L)	5	2.5	5	2.5	2.5	2.5	2.5

### 3.3 Prodigiosin inhibited biofilm formation and viability of biofilm cells

**Figure 3:**
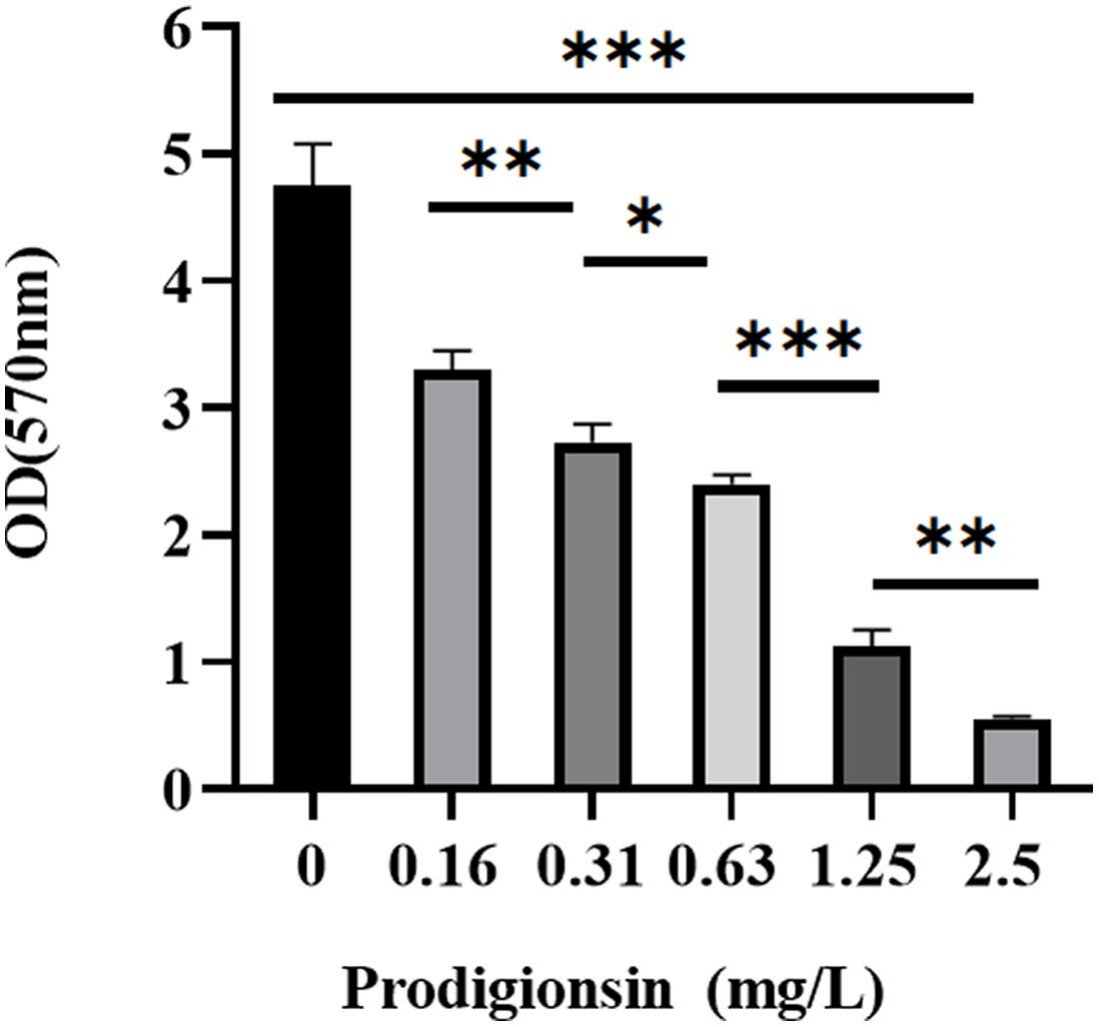
Dosage effects of prodigiosin on the biofilm biomass of MRSA USA300. Bacteria were cocultured in TSB containing 1% glucose (TSBG) with prodigiosin (0, 0.16, 0.31, 0.63, 1.25 or 2.5 mg/L) in a 96-well plate at 37°C for 24 h. Biofilm was stained by 0.1% crystal violet (m/V) for 20 min, and OD570 values were measured after 95% ethanol dissolved the staining solution. The sample treated without prodigiosin adding served as the control. Data were expressed as mean ± SEM (*n* = 3). ^***^p < 0.001, ^**^p < 0.01, and ^*^p < 0.05, compared to the control.

**Figure 4:**
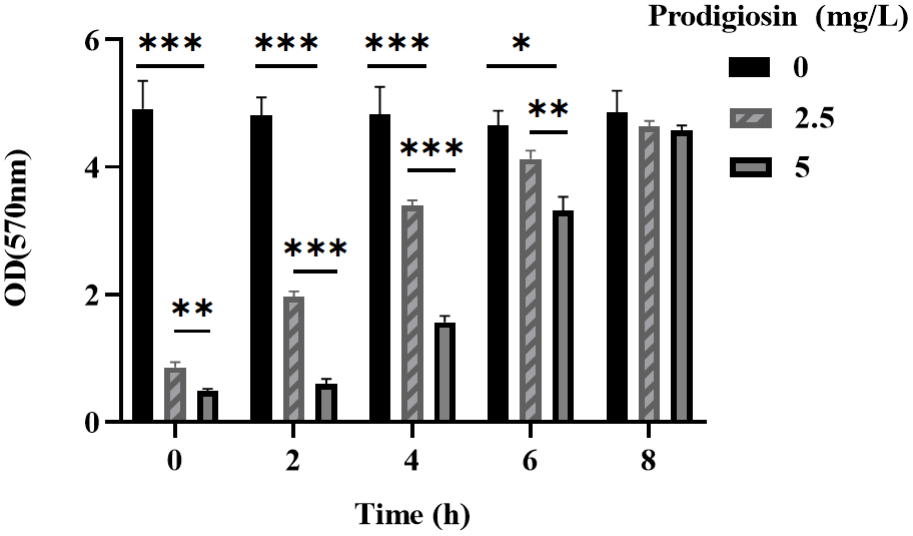
Addition time effects of prodigiosin on the biofilm biomass of MRSA USA300. Bacteria were cultured with TSBG in a 96-well plate at 37°C. Prodigiosin was added at concentrations of 2.5 mg/mL or 5 mg/mL every 2 h from the start of cultivation (0 h) to 8 h, and bacteria were cultured up to 24 h. OD570 values were measured to indicate the biomass of biofilm. Data were expressed as mean ± SEM (*n* = 3). ^***^p < 0.001, ^**^p < 0.01, and ^*^p < 0.05, compared to the control.

The effects of the different prodigiosin concentrations and addition times on biofilm formation were investigated as shown in Figures 3, 4. The results showed that early prodigiosin addition had a greater inhibitory effect against biofilm formation, analogous to the observed effect on bacterial growth.

**Figure 5:**
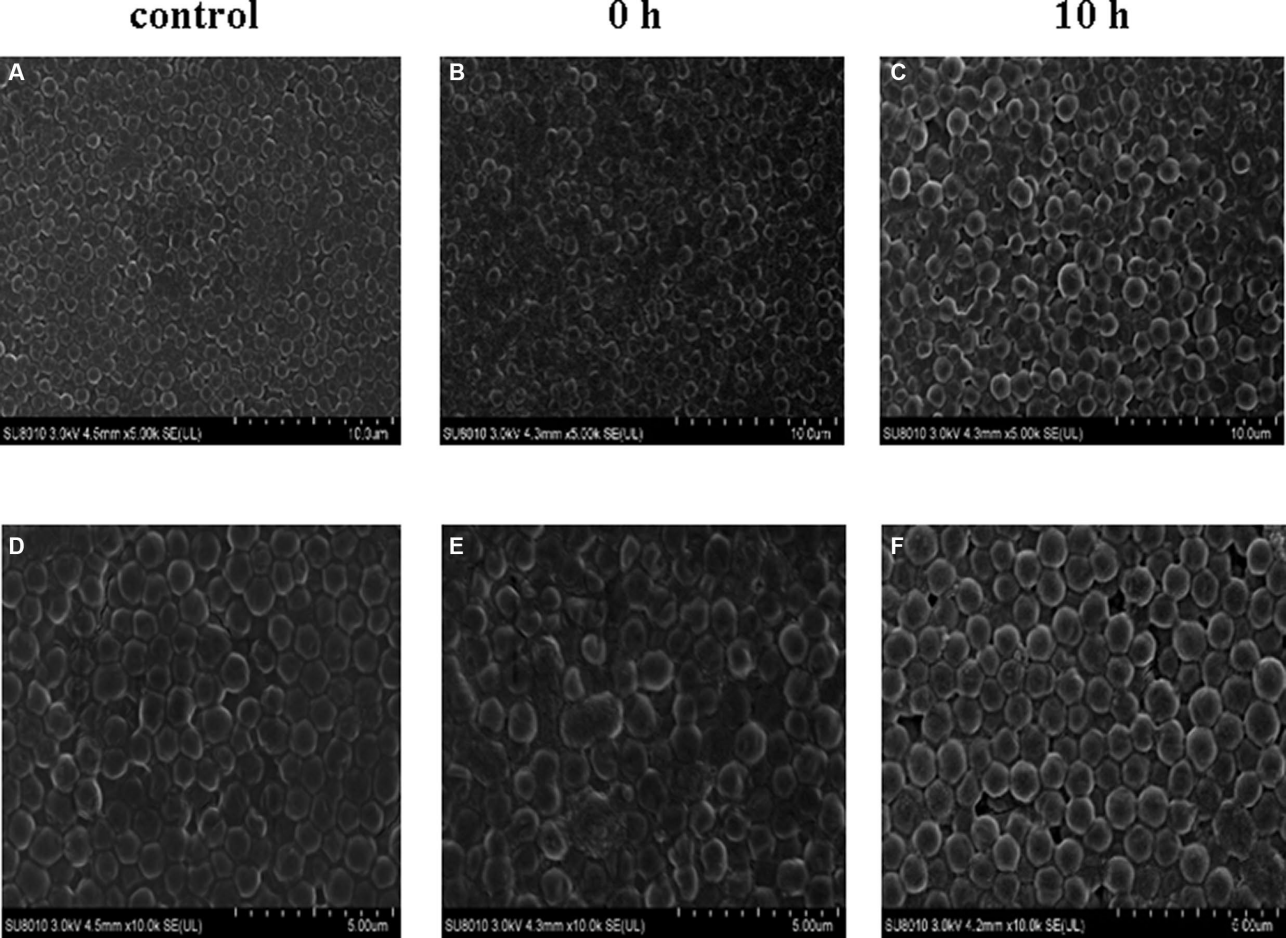
Prodigiosin inhibitory effects on the formation of MRSA USA300 biofilm assessed by scanning electron microscopy. Bacteria were cultured with TSBG in a 12-well plate at 37°C. **(A,C)**: 0 mg/L prodigiosin; **(B,D)**: 2.5 mg/L prodigiosin added at 0 h of cultivation; **(E,F)**: 2.5 mg/L prodigiosin added at 10 h of the cultivation; bacteria were cultured up to 24 h and biofilms obtained were washed with PBS and fixed with 2.5% glutaraldehyde at 4°C for 1 h. **(A–C)** Scale bar: 10.0 μm. **(D–F)** Scale bar: 5.00 μm.

In addition, we conducted a SEM analysis of biofilm formation of *S. aureus* treated with 2.5 mg/L prodigiosin at the beginning of bacterial growth (0 h) and in the stationary phase (10 h) in TSBG for biofilm formation (Figure 5). In the control group, the cells were closely arranged, with each cell having an intact, round edge without wrinkles and breaks (Figures 5A,D). The same phenomenon was observed for cells treated with 2.5 mg/L prodigiosin in the stationary phase (10 h) (Figures 5C,F). By contrast, the cells became wrinkled, severely deformed and broken following treatment with 2.5 mg/L prodigiosin at the beginning of bacterial growth (0 h) (Figures 5B,E).

**Figure 6:**
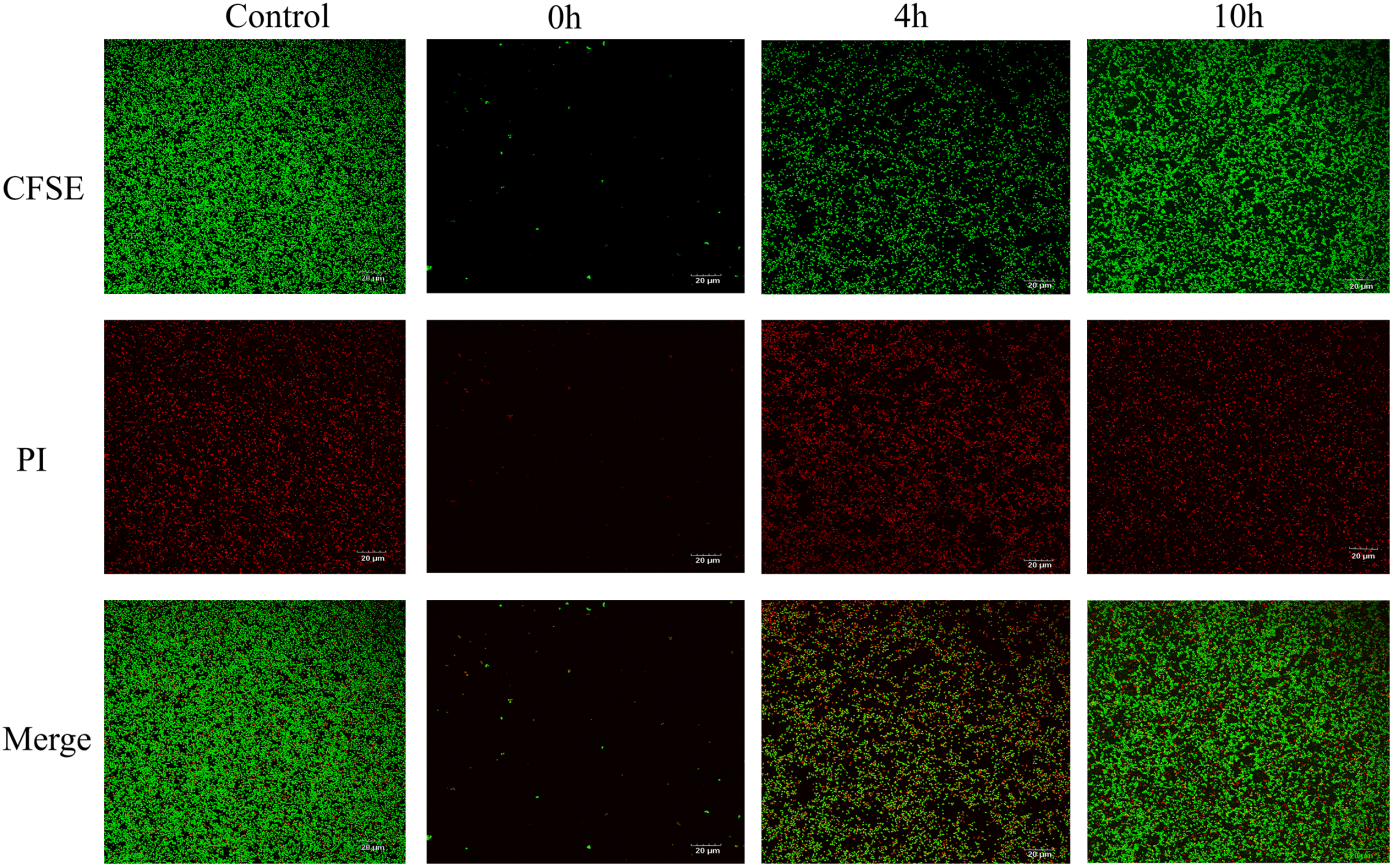
Prodigiosin inhibitory effects on MRSA USA300 biofilm assessed by confocal Laser microscopy. Bacteria were cultured in a 12-well plate with the glass coverslips at 37°C, and 2.5 mg/L prodigiosin was added at start of cultivation (0 h), 4 h and 10 h respectively, and bacteria were cultivated up to 24 h. Then, biofilm was strained with green fluorescent labeled CFSE for live cell and with red fluorescent PI for dead cell (Ar4 88/20 0–500 nm). Treated without prodigiosin served as control. Scale bar: 20 μm.

While MRSA USA300 formed a strong biofilm under the control conditions according to the CLSM analysis, prodigiosin drastically reduced the fluorescence intensity of live cells in the biofilm at the beginning and in the logarithmic phase (Figure 6). However, when 2.5 mg/L prodigiosin was added in the stationary phase, there was almost no effect on the fluorescence intensity of cells in the biofilm.

**Figure 7:**
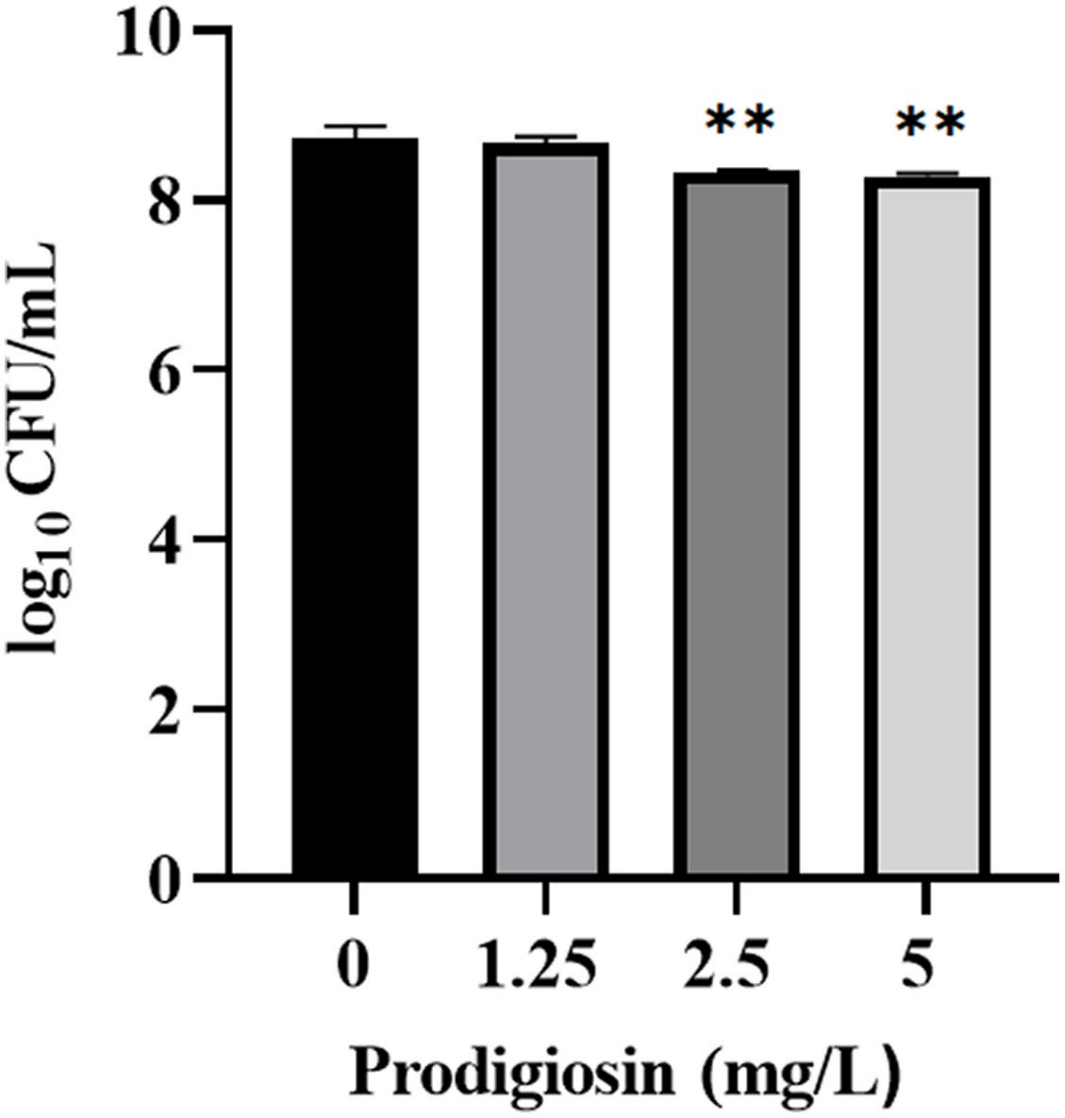
Effect of prodigiosin on the viability of MRSA USA300 cells in the biofilm. Bacterial biofilm (24 h old) was treated with prodigiosin (1.25, 2.5 or 5 mg/mL) for 24 h. Viability of the cells was measured by CFU counting. The sample treated without prodigiosin served as the control. Data were expressed as mean ± SEM (*n* = 3). ^**^p < 0.01, compared with control.

The effects of prodigiosin on the viability of cells in the biofilm was measured by CFU counting (Figure 7). The results showed that 2.5 mg/Land 5 mg/L prodigiosin significantly inhibited the viability of bacterial cells in the biofilm, but there is no significant difference between the two concentrations of prodigiosin.

### 3.4 General features of the transcriptome

**Figure 8:**
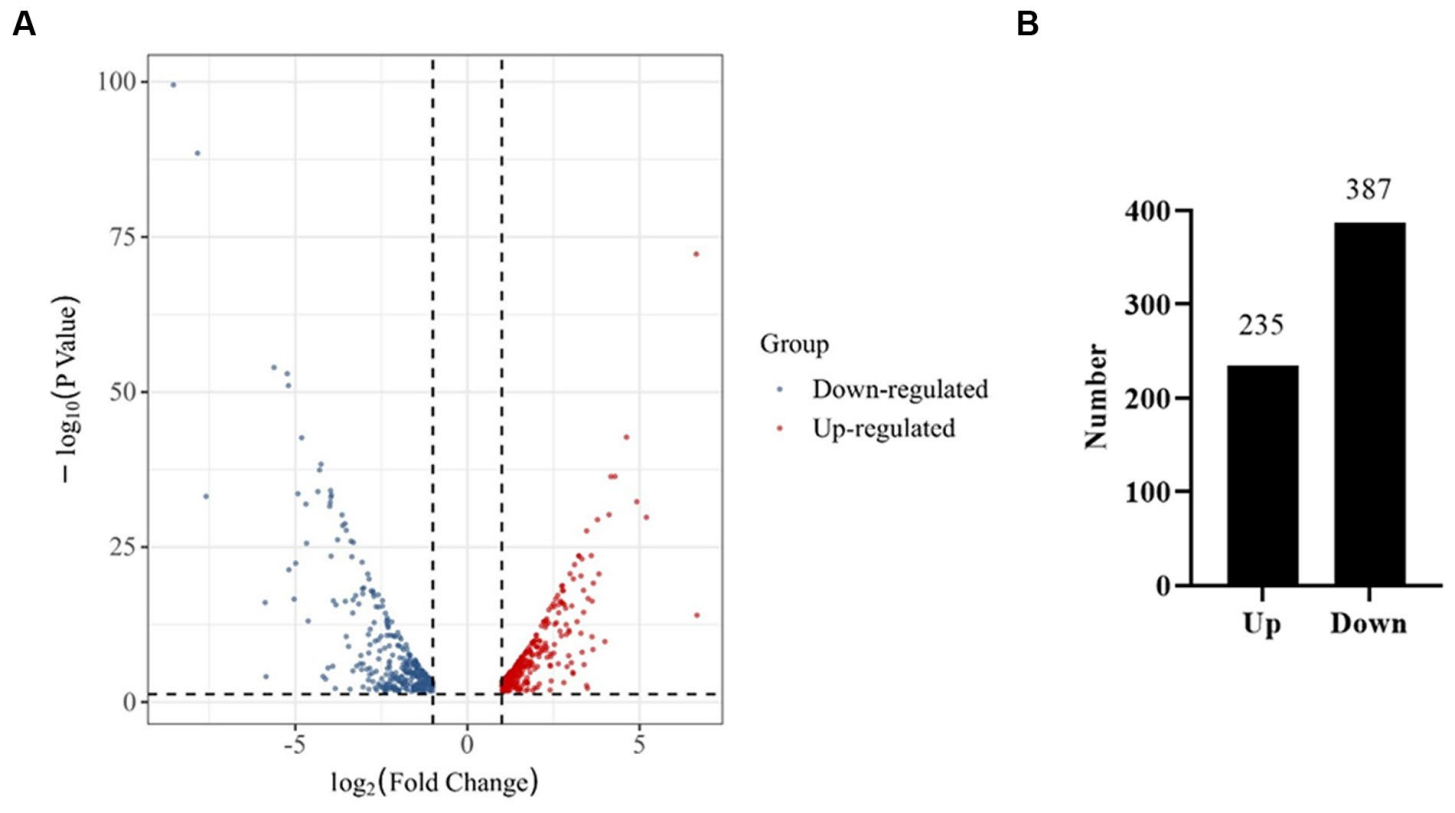
RNA-Seq analysis of genes expressed in the control and prodigiosin treatment group. **(A)** Volcano plot showing the differentially expressed genes (DEGs) in MRSA USA300 cells treated with 1/8 MIC (0.31 mg/L) prodigiosin for 5 h. **(B)** Quantitive comparison of the up- and down-regulated genes.

**Figure 9:**
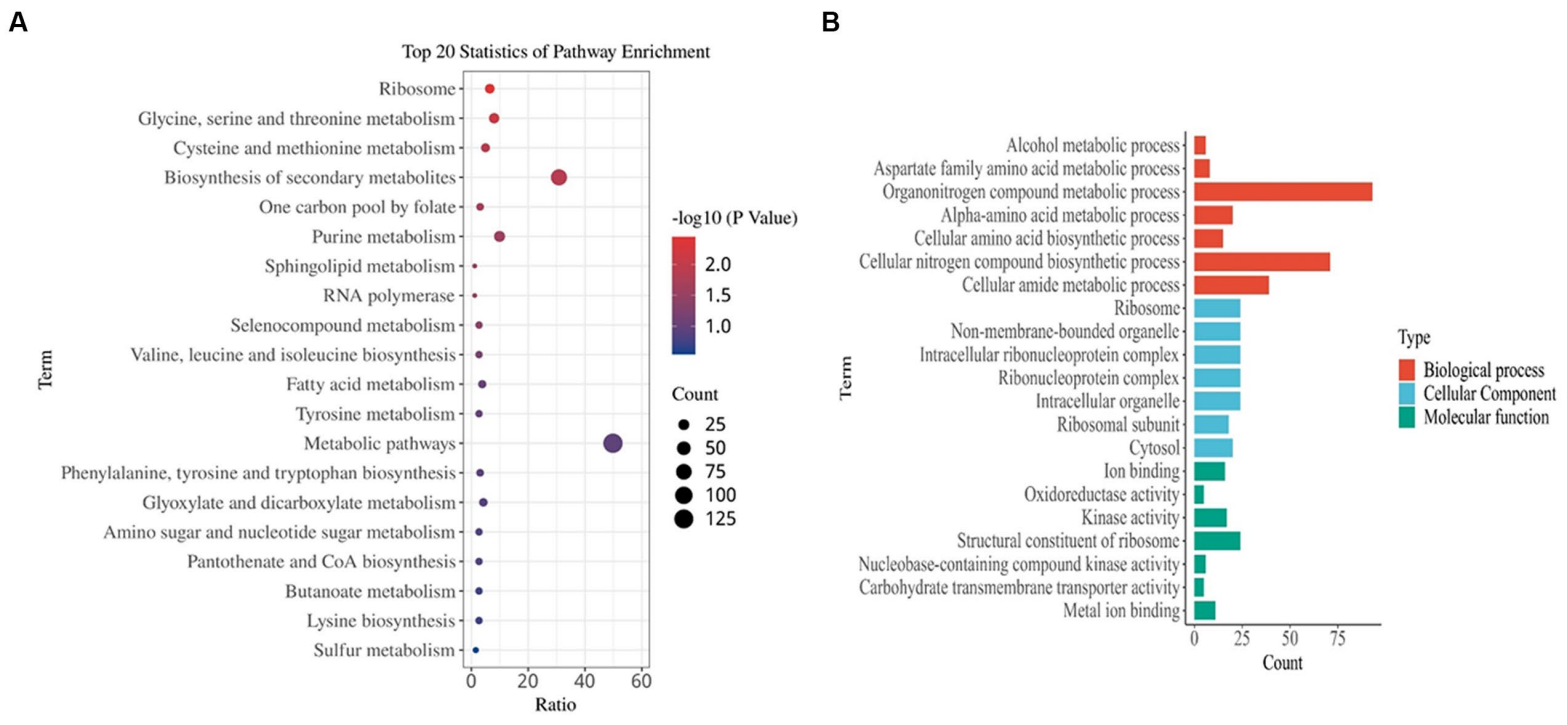
Functional enrichment analysis of DEGs. **(A)** Kyoto Encyclopedia of Genes and Genomes (KEGG) pathway analysis. **(B)** Gene ontology (GO) analysis. The results are summarized in the following three main categories: biological process, molecular function, and cellular component. DEGs were obtained by comparing the treated MRSA USA300 (5 h post 1/8 MIC prodigiosin) and non-treated MRSA USA300 groups.

A comparatively low concentration of 0.31 mg/L of prodigiosin (1/8 MIC) significantly inhibited biofilm formation when it was added at the beginning of the culture period (Figure 3). Therefore, gene expression was investigated by transcriptome analysis to explain the underlying molecular mechanism. Based on an adjusted |log2(fold change)| ≥1 and *q*-value ≤0.05, 622 DEGs were identified in the treated group, 235 of which were up- and 387 downregulated compared to the control (Figure 8). Then, KEGG pathway enrichment analysis was conducted to further understand the involved pathways (Figure 9A). In addition, Gene Ontology (GO) enrichment analysis revealed three specific categories with 21 significantly enriched GO terms (Figure 9B).

### 3.5 Differentially expressed genes related to the cell envelope

The most significant DEGs are listed in the Supplementary Table S4. The genes encoding *N*-acetylglucosamine-1-phosphate uridyltransferase (*glmU*), N-acetylmuramic acid 6-phosphate etherase (*murQ*), N-acetylglucosamine-6-phosphate deacetylase (*nagA*) and N-acetylmuramoyl-L-alanine amidase (*aaa*) were significantly downregulated. The upregulated genes included those encoding cell wall damage response proteins, such as the two-component system DNA-binding response regulator VraR, D-alanyl-lipoteichoic acid biosynthesis protein DltD and D-alanyl-lipoteichoic acid biosynthesis protein DltB, as well as the 9 capsular polysaccharide biosynthesis proteins (Supplementary Table S4). The genes encoding MSCRAMMs were significantly downregulated, including *sdrC* encoding serine-aspartate repeat-containing protein C, *sdrD* encoding serine-aspartate repeat protein D, spa encoding surface protein A, *ebpS* encoding elastin-binding protein S, as well as the three genes *ecb*, *scb* and *efb* encoding fibrinogen-binding protein. The three genes encoding the immunoglobulin G-binding protein Sbi, as well as immunodominant antigen A and B (*isaA* and *isaB*) related to immunity were also significantly downregulated, as were the genes encoding iron-regulatory proteins including *srtA* and *scdA*. The key genes for arginine synthesis, *arcC* encoding carbamate kinase and *argF* encoding ornithine carbamoyltransferase, were also significantly downregulated. In addition, the three genes *nreA*, *nreB* and *srrB* encoding a quorum sensing sensor histidine kinase were also downregulated significantly. The genes encoding the other surface proteins were upregulated, including *sdrE* (serine-aspartate repeat protein E) and *sasA* (adhesin).

### 3.6 RT-PCR validation

**Figure 10:**
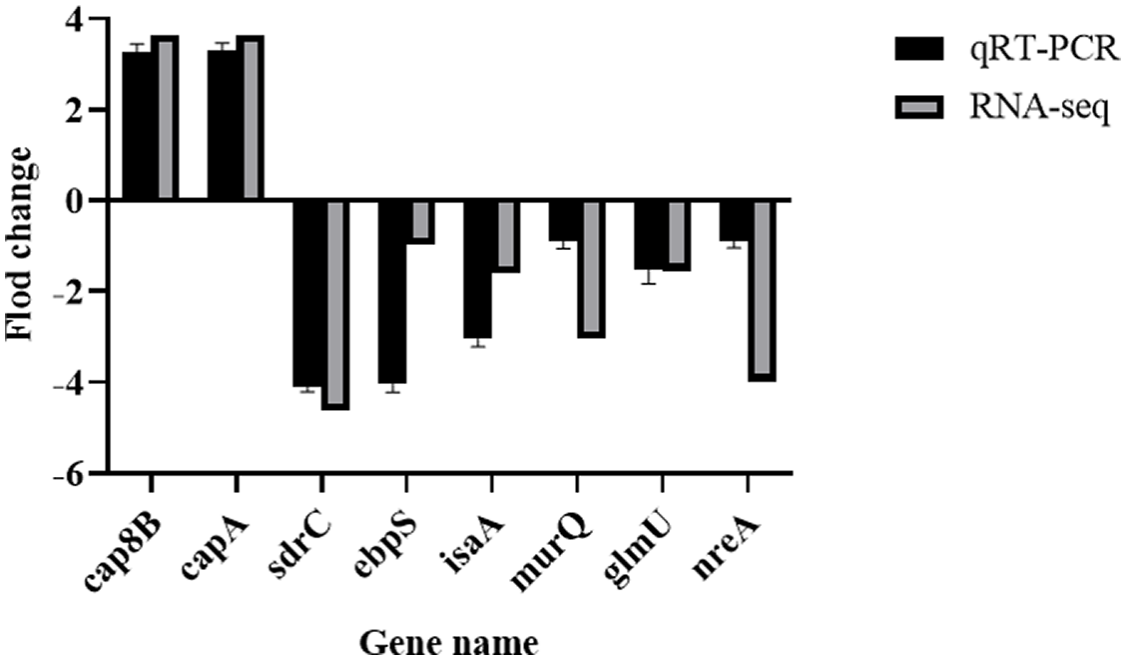
Validation of RNA sequencing data by RT-PCR. MRSA USA300 was exposed to 1/8 MIC prodigiosin for 5 h. the expression levels of eight genes related to the cell envelope biosynthesis were confirmed by RT-PCR. Data were expressed as mean ± SEM (*n* = 3).

The expression levels of 8 randomly selected DEGs were consistent with the RNA-Seq results according to the qRT-PCR assay (Figure 10). This result confirmed the validity of the RNA-seq data.

## 4 Discussion

In recent years, the threat caused by antibiotic-resistant bacteria to people’s health has become increasingly serious. Currently, the main solution to this problem is the combination of antibiotics (David et al., 2019; Nisarg et al., 2020; Yan et al., 2023). However, the discovery of new antibacterial agents is still the most promising way to overcome antibiotic resistance. Prodigiosin has been found to have significant bacteriostatic effects on many pathogens (Wang B. et al., 2022; Wang Y. J. et al., 2022).

In this study, the MIC value of prodigiosin ranged from 2.5 to 5 mg/L for all *S. aureus* isolates. This was inconsistent with previously reported MIC values, which were in the range of 0.25–32 mg/L (Ji et al., 2015; Danevčič et al., 2016b; Suryawanshi et al., 2017; Wang B. et al., 2022; Wang Y. J. et al., 2022; Yan et al., 2023). Possible reasons for the wide range of MIC values reported in the literature may be prodigiosin samples obtained from different strains, different purification methods, as well as different methods for measuring MIC values and different testing strains. By contrast, Wang B. et al. (2022) and Wang Y. J. et al. (2022) reported that MSSA and MRSA strains had the same MIC values. However, the results of Yip et al. (2021) indicated that 10 mg/L prodigiosin was necessary to inhibit the growth of MSSA, while an even higher concentration was necessary to inhibit the growth of MRSA. This difference between MSSA and MRSA strains merits further study in the future.

The results of crystal-violet staining indicated that prodigiosin significantly inhibited biofilm formation, and at a concentration of 1.25 mg/L, the inhibition rate reached 76.24%. Similarly, a previous study indicated that prodigiosin could inhibit *Pseudomonas aeruginosa* biofilm formation by inducing the production of reactive oxygen species (Kimyon et al., 2016). Moreover, prodigiosin inhibited biofilm formation in MRSA ATCC 43300 and had synergistic activity with vancomycin (Yan et al., 2023). However, another study showed that prodigiosin actually induced biofilm formation in *P. aeruginosa*, while decreasing biofilm formation in *Enterococcus faecalis*, *Salmonella enterica* serovar typhimurium and *E. coli* (Yip et al., 2021).

Two component signal regulatory systems (TCSs) are composed of a histidine kinase that acts as a membrane sensor and a response regulator, which can receive the information from the kinase and mediate the relevant intracellular response. Notably, studies have shown that biofilm formation is also regulated by two-component regulatory systems. Our results indicated that three pathways of TCSs in *S. aureus* were differentially expressed, including one involved in cell wall biosynthesis (VraR), one associated with the sensor histidine kinase genes *nreA*, *nreB* and *srrB* that respond to oxygen availability, and another involved in autolysis (LytS).

Peptidoglycan is an important component of the cell wall of Gram-positive bacteria, providing rigidity and maintaining the shape of cells. GlmU as a bifunctional enzyme that converts GlcNAc-1-P and UTP into UDP-GlcNAc for peptidoglycan biosynthesis (Vithani et al., 2014; Soni et al., 2023). Some researchers reported that GlmU may be a promising target for inhibiting the growth of pathogenic bacteria (Mochalkin et al., 2008; Purushotham et al., 2019; Palathoti and Azam, 2023). Here, we report the first evidence that GlmU expression can be inhibited in MRSA using prodigiosin.

MurQ converts N-acetyl-D-glucosamine 6-phosphate and I-lactate to form N-acetylmuramate 6-phosphate, and NagA converts N-acetyl-D-glucosamine 6-phosphate to produce D-glucosamine 6-phosphate in the peptidoglycan pathway. In addition, Aaa hydrolyzes the link between N-acetylmuramoyl and L-amino acid residues in some cell-wall glycopeptides during the recycling of peptidoglycan. Hadi et al. (2013) reported that MurQ from *Haemophilus influenzae* can bind an inhibitor, whereas Stephanie et al. (2022) reported that a defect of cell wall recycling conferred antibiotic resistance to *S. aureus*, due to partial collapse of a pathway involving MurQ. Further research is needed to determine whether prodigiosin binds to MurQ and regulate the synthesis of peptidoglycans by molecular docking and experimental verification.

Our results indicated that prodigiosin significantly inhibited most cell wall anchor proteins related to biofilm formation, including SdrC, SdrD, Sbi, IsaA, IsaB, Efb, Ecb, Scb and SarX, which was consistent with the literature (Geoghegan et al., 2013; Ma et al., 2022; Zhang et al., 2022). Similarly, SdrC, SdrD and Sbi were found to be inhibited by lomitapide, which also decreases biofilm formation (Zhang et al., 2022). SdrC is engaged in the first stage of adhesion among cells by low-affinity bonds, and also promotes the adherence of cells to surfaces, which may contribute to biofilm formation (Pi et al., 2020). SdrD is a cell surface protein that plays a crucial role in the attachment of *S. aureus* to the extracellular matrix (ECM) during biofilm formation (Salinas et al., 2022). Accordingly, the attachment and biofilm formation of MRSA was inhibited by reducing the expression of the *sdrD* gene and increasing the distance between the ECM-interacting domain and the bacterial surface (Iwata et al., 2021).

Similarly, disruption of the *isaA* virulence factor gene decreased biofilm formation (Ma et al., 2022). Mackey-Lawrence et al. (2009) showed that IsaB exhibited higher affinity for double-stranded DNA than single-stranded DNA and RNA, but it was found that IsaB did not contribute to biofilm formation. Further studies are necessary to determine whether IsaB influences biofilm formation in the MRSA USA300 strain and what role it may play in the establishment and/or progression of *S. aureus* infection.

Fibronectin/fibrinogen-binding proteins (Ecb, Scb and Efb) are expressed by the majority of *S. aureus* strains, in which they facilitate the colonization of biotic surfaces and can promote biofilm matrix formation via a mechanism based on Zn^2+^-dependent, low affinity bonds between adjacent cells (Geoghegan et al., 2013). In a previous study, the genes encoding fibrinogen binding proteins involved in the biosynthesis of polysaccharide intercellular adhesin (PIA) were found to be downregulated when MRSA was treated with Manuka honey (Kot et al., 2020), which is consistent with our results.

Biofilm production by *S. aureus* was promoted by the *sarX* gene, which was attributed to increased expression of the *ica* operon and PIA. Furthermore, deletion of *sarX* reduced *S. aureus* biofilm formation by decreasing *ica* operon expression and PIA biosynthesis as well as downregulating *spa* (Hao et al., 2021). This result was in agreement with our research, which showed that the expression of sarX and spa were downregulated when prodigiosin reduced biofilm formation. The results of Ma et al. (2022) indicated that the adhesin/biofilm-related genes and hemolysin genes, such as *sarX* and *hlgC*, were simultaneously downregulated with *isaA*, and the same phenomenon was observed in our study.

The genes encoding cell wall synthesis related proteins including DltB, DltD, 9 capsular polysacchar-ide biosynthesis proteins and VraR were upregulated according to bioinformatics analysis. This was consistent with earlier reports, which indicated that VraR expression was induced by cell wall antimicrobials, and the cell wall of *S. aureus* was damaged by lomitapide (Yin et al., 2006; Levinger et al., 2012; Zhang et al., 2022).

**Figure 11:**
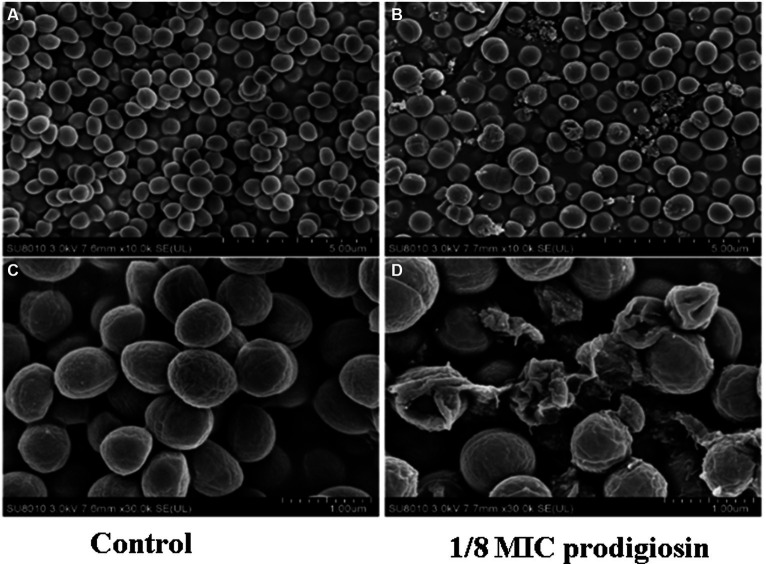
Prodigiosin effects on cell structure of MRSA USA300 assessed by transmission electron microscopy. Bacteria were cocultured with 1/8 MIC (0.31 mg/L) prodigiosin using 5 mL TSBG in 50 mL centrifuge tubes at 37°C for 12 h. And the cells were collected and fixed with 2.5% glutaraldehyde at 4°C overnight. The fixed cells were observed on a H-7650 TEM. **(A,C)** Treated with 0 mg/L prodigiosin. **(B,D)** Treated with 1/8 MIC (0.31 mg/L) prodigiosin. **(A,B)** Scale bar: 5.00 μm. **(C,D)** Scale bar: 1.00 μm.

The genes *nreA*, *nreB* and *srrB* encode sensor histidine kinases of two-component systems related to quorum sensing that are located on the cell membrane. Our SEM and TEM results showed that prodigiosin destroyed the cell membrane integrity of *S. aureus* (Figures 5, 11). Consistently, earlier reports indicated that the principal antibacterial mechanism of prodigiosin is based on the disruption of the cell membrane (Danevčič et al., 2016b; Suryawanshi et al., 2017; Herráez et al., 2019; Wang B. et al., 2022; Wang Y. J. et al., 2022). Danevčič et al. (2016b) reported that prodigiosin damaged the cytoplasmic membrane of *B. subtilis* and increased its permeability, which is associated with autolysin biosynthesis. The same research team found that cells treated with prodigiosin at a dose higher than the MIC had a leaky cell membrane, but there was no significant disintegration (Danevčič et al., 2016a). Further evidence provided by Suryawanshi et al. (2017) indicated that the surface of *S. aureus* treated by prodigiosin was heterogeneous. These findings suggested that prodigiosin acts as an external stressor able to damage the plasma membrane (Danevčič et al., 2016b). NreB is a cytoplasmic protein with four conserved cysteine residues that comprise an iron-sulfur cluster (Annegret et al., 2004; Sangare et al., 2020). The downregulation of the iron-sulfur cluster repair protein ScdA may further decreased the content of iron-sulfur clusters. It is speculated that the decrease of iron-sulfur clusters reduced biofilm formation, indicating that iron-sulfur cluster proteins may be an important target of prodigiosin. However, their role in biofilm formation needs further study. In addition, Đukanović et al. (2022) reported that emodin from *Frangula* bark inhibited biofilm formation in *S. aureus* by downregulating the expression of the *srrB* gene, which is an important target for inhibiting biofilm formation by reducing the biosynthesis of adhesion. These earlier reports are consistent with our results.

It was also reported that pneumococcal LytS is involved in anchoring both the capsular polysaccharide and teichoic acids to cell wall, which is important for virulence (Ye et al., 2018; Huang et al., 2021). Due to their lytic activities these proteins are related to the synthesis of the cell wall, autolysis and release of genomic DNA, which eventually become the biofilm matrix, thus promoting biofilm formation. However, the function of LytS in biofilm formation by *S. aureus* needs further study.

Arginine regulates the expression of downstream genes in the two-component system after phosphorylation (Fan et al., 2020), including those involved in biofilm synthesis. The genes *arcC* and *argF* involved in the biosynthesis of arginine were downregulated when the biofilm formation of MRSA USA300 was significantly reduced by prodigiosin. Samaneh et al. (2022) reported that L-arginine supplementation enhanced biofilm formation in *S. mutans* at concentrations of 5 and 10 μM. Similarly, Liu et al. (2022) found that arginine could promote biofilm formation in *P. aeruginosa*. Moreover, Adhar et al. (2022) reported that the small regulatory RNA Teg58 influenced arginine biosynthesis genes (i.e., *argGH* encoding arginosuccinate synthetase and lyase) to reduce biofilm formation. However, Zhu et al. (2007) indicated that the catabolic pathway of arginine did not play a significant role in the inhibition of biofilm formation in *S. aureus*. Nevertheless, Qu et al. (2020) showed that the novel coumarin derivative DCH inhibited MRSA biofilm formation by targeting a repressor of the arginine catabolic pathway. Further studies are needed to clarify if the downregulation of *arcC* and *argF* influences the yield of arginine, as well as to evaluate the effects of prodigiosin on arginine synthesis and biofilm formation.

## 5 Conclusion

Our results showed that prodigiosin can affect the cell envelope formation, including the destruction of the cell wall and cell membrane structures, as well as the formation of biofilm. At a concentration of 2.5 mg/L, prodigiosin also significantly inhibited the vitality of MRSA cells in the biofilm. Transcriptomic analysis of the stress response of MRSA USA300 exposed to 1/8 MIC (0.31 mg/L) prodigiosin indicated that three TCSs were differentially expressed, including one associated with autolysis (transcriptional regulator LytS), one controlling virulence genes (*nreA*, *nreB* and *srrB*) involved in quorum sensing related to oxygen availability, and another involved in cell wall biosynthesis (*vraR*, *murQ* and *glmU*). In addition, MSCRAMMs including SdrC, SdrD, Spa, Efb, etc. as well as the key arginine synthesis enzymes (ArcC and ArgF) were also affected. This evidence expands the knowledge of the response of MRSA USA300 to prodigiosin, providing a theoretical basis for designing highly effective inhibitors for the treatment of multidrug-resistant MRSA bacteria.

## Data availability statement

The datasets presented in this study can be found in online repositories. The names of the repository/repositories and accession number(s) can be found in the article/Supplementary material.

## Author contributions

XL: Writing – original draft, Writing – review & editing, Conceptualization, Data curation, Formal analysis, Funding acquisition, Investigation, Methodology, Supervision. ZW: Data curation, Formal analysis, Methodology, Resources, Writing – original draft. ZY: Methodology, Resources, Writing – review & editing. WW: Data curation, Methodology, Resources, Writing – review & editing. YW: Resources, Writing – review & editing. WWu: Data curation, Software, Writing – review & editing. YP: Data curation, Resources, Writing – review & editing. SZ: Writing – review & editing. YY: Software, Writing – review & editing. JZ: Conceptualization, Formal analysis, Supervision, Writing – review & editing.
