# Supplementary material for: Transcriptomic analysis of cell envelope inhibition by prodigiosin in methicillin-resistant Staphylococcus aureus
Source: Front Microbiol. 2024 Jan 22;15:1333526. doi: 10.3389/fmicb.2024.1333526 (PMC10839101; doi:10.3389/fmicb.2024.1333526)
Supplement: Supplementary file 2 [file Table_1.docx]

**Supplementary Table Captions**

**Supplementary Table** 1 the sequences of primer of the gene identified

**Supplementary Table** 2 Identification and sources of clinical isolates of *S. aureus*

**Supplementary Table** 3 Antibiotic spectrum data of *S. aureus* strains

**Supplementary Table** 4 Important differential expression genes between PG-treated *S. aureus* (USA 300_EG) and non-treated S. aureus (USA 300_CK) groups

**Supplementary Table** 1 the sequences of primer of the gene identified

| Name | Sequence（5’to 3’） |
| --- | --- |
| cap8B forward | TGCGTAAGCCAACACAAAACT |
| cap8B reverse | GGTACAGGGCCAGCTGTTAG |
| capA forward | AAGCAGACGGTACAGCAGTT |
| capA reverse | AGCGCGACAACTAATCCTAA |
| sdrC forward | GCAGGTAAACATACTCCGGCT |
| sdrC reverse | AATGCCGCGAATAATCCACC |
| ebpS forward | ACTCGACTGAGGATAAAGCGT |
| ebpS reverse | GGCATGTGGTTTTGAAGCGG |
| isaA forward | TCATGGCTCAACGTACTGGTGTTTC |
| isaA reverse | CCTGAAGCACCTGATGGGTTGTAAG |
| murQ forward | GCAAGTCCCGTTAGCAGT |
| murQ reverse | GAGGGTCAGTATTGAATGTAGG |
| glmU forward | GATGGTGCCAAGGTTTCA |
| glmU reverse | CTTTGCCGACGATAGTTT |
| nreA forward | TCGCTTTCTATGAATCTAAC |
| nreA reverse | GCTATTACCATACGCTTTCC |
| 16S rRNA forward | GACGGTCTTGCTGTCACTTA |
| 16S rRNA reverse | AGTTCCAGTGTGGCCGATCA |

**Supplementary Table** 2 Identification and sources of clinical isolates of *S. aureus*

| Numer | Isolate number | Date of sampling | Source/origin | Bacterial identity^a^ | probability of identity^b^ |
| --- | --- | --- | --- | --- | --- |
| 1 | 21612804 | 2021/4/15 | pus | *S. aureus* |  |
| 2 | 21612346 | 2021/4/12 | pus | *S. aureus* |  |
| 3 | 19828420 | 2019/10/6 | blood | *S. aureus* |  |
| 4 | 21610437 | 2021/3/26 | pus | *S. aureus* | 99% |
| 5 | 21609490 | 2021/3/19 | wound surface | *S. aureus* |  |
| 6 | 21607345 | 2021/3/2 | pus | *S. aureus* |  |
| 7 | 21600164 | 2021/1/2 | drainage fluid | *S. aureus* |  |
| 8 | 21609392 | 2021/3/18 | pus | *S. aureus* |  |
| 9 | 21604053 | 2021/2/1 | pus | *S. aureus* |  |
| 10 | 20639244 | 2020/12/15 | pus | *S. aureus* |  |
| 11  12  13  14  15  16  17  18  19  20  21  22  23  24  25  26  27  28  29  30  31  32  33  34  35  36  37  38  39  40  41  42 | 20640432  20637209  21608485  20638708  21610333  20634485  21608328  21631084  20637883  20632640  20632641  20632883  20633115  20633301  20634749  20634915  20635041  20635052  20635070  20635135  20635151  20635502  20635935  20636352  20636769  20637031  20637224  20637880  20638024  20638594  20639877  20639989 | 2020/12/24  2020/11/29  2021/3/11  2020/12/11  2021/3/25  2020/11/7  2021/3/10  2021/9/16  2020/12/4  2020/10/24  2020/10/24  2020/10/27  2020/10/28  2020/10/30  2020/11/10  2020/11/11  2020/11/11  2020/11/12  2020/11/12  2020/11/12  2020/11/12  2020/11/16  2020/11/19  2020/11/22  2020/11/26  2020/11/28  2020/11/29  2020/12/5  2020/12/6  2020/12/10  2020/12/21  2020/12/21 | drainage fluid  drainage fluid  pus  pus  wound surface  pus  catheter  pus  pus  [sputum](javascript:;)  [sputum](javascript:;)  [throat swab](javascript:;)  [throat swab](javascript:;)  [throat swab](javascript:;)  [throat swab](javascript:;)  [sputum](javascript:;)  [sputum](javascript:;)  [throat swab](javascript:;)  [throat swab](javascript:;)  [nose swab](javascript:;)  [sputum](javascript:;)  [throat swab](javascript:;)  [throat swab](javascript:;)  [sputum](javascript:;)  [sputum](javascript:;)  [sputum](javascript:;)  [throat swab](javascript:;)  Pus  [nose swab](javascript:;)  [sputum](javascript:;)  [sputum](javascript:;)  [sputum](javascript:;) | *S. aureus*  *S. aureus*  *S. aureus*  *S. aureus*  *S. aureus*  *S. aureus*  *S. aureus*  *S. aureus*  *S. aureus*  *S. aureus*  *S. aureus*  *S. aureus*  *S. aureus*  *S. aureus*  *S. aureus*  *S. aureus*  *S. aureus*  *S. aureus*  *S. aureus*  *S. aureus*  *S. aureus*  *S. aureus*  *S. aureus*  *S. aureus*  *S. aureus*  *S. aureus*  *S. aureus*  *S. aureus*  *S. aureus*  *S. aureus*  *S. aureus*  *S. aureus* |  |
| 43  44  45  46  47  48  49  50  51  52  53  54  55 | 20640748  20640937  20641083  19636371  21600095  21607290  21609651  21610023  21610258  21611144  21611240  21611337  21612941 | 2020/12/27  2020/12/29  2020/12/30  2019/9/11  2021/1/1  2021/3/2  2021/3/21  2021/3/24  2021/3/25  2021/4/2  2021/4/3  2021/4/4  2021/4/17 | [sputum](javascript:;)  [throat swab](javascript:;)  [sputum](javascript:;)  [arthroedema](javascript:;)  [sputum](javascript:;)  [throat swab](javascript:;)  [sputum](javascript:;)  [throat swab](javascript:;)  ear swab  [sputum](javascript:;)  [throat swab](javascript:;)  [nose swab](javascript:;)  Pus | *S. aureus*  *S. aureus*  *S. aureus*  *S. aureus*  *S. aureus*  *S. aureus*  *S. aureus*  *S. aureus*  *S. aureus*  *S. aureus*  *S. aureus*  *S. aureus*  *S. aureus* |  |

^a^MALIDA-TOF-MS

^b^ The confidence level is 99% on probability of identity (%)

**Supplementary Table** 3 Antibiotic spectrum data of *S. aureus* strains

|  | G^a^ | CN^b^ | MXF^c^ | LVX^d^ | CLI^e^ | LZD^f^ | QD^g^ | TGC^h^ | OX^i^ | RD^j^ | CIP^k^ | SMZ-TMP^l^ | EM^m^ | VA^n^ | TE^o^ |
| --- | --- | --- | --- | --- | --- | --- | --- | --- | --- | --- | --- | --- | --- | --- | --- |
| 21612804 | R | S | S | S | S | S | S | S | R | S | S | S | S | S | S |
| 21612346 | R | S | S | S | S | S | S | S | R | S | S | S | S | S | S |
| 19828420 | R | S | S | S | R | S | S | S | R | S | S | S | R | S | S |
| 21610437 | R | S | S | S | R | S | S | S | R | S | S | S | R | S | S |
| 21609490 | R | S | R | R | R | S | S | S | R | S | R | S | R | S | S |
| 21607345 | R | S | S | S | S | - | S | S | R | S | S | S | S | S | S |
| 21600164 | R | S | S | S | R | S | S | S | R | S | S | R | R | S | S |
| 21609392 | R | S | S | S | S | S | S | S | R | S | I | S | S | S | S |
| 21604053 | R | S | R | R | S | S | S | S | R | S | R | S | S | S | S |
| 20639244 | R | S | S | S | R | S | S | S | R | S | S | S | R | S | S |
| 20640432 | R | S | S | S | R | S | S | S | R | S | S | S | R | S | S |
| 20637209 | R | S | S | S | R | S | S | S | R | S | S | S | R | S | S |
| 21608485 | R | R | R | R | R | S | S | S | R | S | R | S | R | S | R |
| 20638708 | R | S | S | S | R | S | S | S | R | S | S | S | R | S | S |
| 21610333 | R | S | S | S | S | S | S | S | R | S | S | S | S | S | S |
| 20634485 | R | S | S | S | R | S | S | S | R | S | S | S | R | S | S |
| 21608328 | R | S | S | S | R | S | S | S | R | S | S | S | R | S | S |
| 21631084 | R | S | S | S | S | S | S | S | R | S | S | S | S | S | S |
| 20637883 | R | S | S | S | S | S | S | S | R | S | S | S | S | S | S |
| 20632640 | R | S | S | S | S | S | S | S | R | S | S | S | S | S | S |
| 20632641 | R | S | S | S | S | S | S | S | R | S | S | S | S | S | S |
| 20632883 | R | R | R | R | R | S | S | S | R | S | R | R | R | S | R |
| 20633115 | R | S | S | S | R | S | S | S | R | S | S | S | R | S | R |
| 20633301 | R | S | S | S | S | S | S | S | R | S | S | S | R | S | S |
| 20634749 | S | S | S | S | S | S | S | S | S | S | S | S | S | S | S |
| 20634915 | R | S | S | S | R | S | S | S | R | S | S | S | R | S | S |
| 20635041 | R | S | S | S | S | S | S | S | R | S | S | S | S | S | S |
| 20635052 | R | S | S | S | S | S | S | S | S | S | S | S | R | S | S |
| 20635070 | R | S | S | I | S | S | S | S | R | S | I | S | S | S | S |
| 20635135 | R | S | S | S | S | S | S | S | R | S | S | S | R | S | S |
| 20635151 | R | S | S | S | S | S | S | S | S | S | S | S | R | S | S |
| 20635502 | R | S | S | S | S | S | S | S | R | S | S | S | R | S | S |
| 20635935 | R | S | S | S | R | S | S | S | R | S | I | S | R | S | S |
| 20636352 | R | S | S | S | R | S | S | S | R | S | S | S | R | S | R |
| 20636769 | R | S | S | S | S | S | S | S | R | S | S | S | R | S | S |
| 20637031 | R | S | S | S | R | S | S | S | R | S | S | S | S | S | R |
| 20637224 | R | S | S | I | R | S | S | S | R | S | I | S | R | S | S |
| 20637880 | R | S | S | I | S | S | S | S | R | S | S | S | S | S | S |
| 20638024 | R | S | S | S | S | S | S | S | R | S | S | S | R | S | S |
| 20638594  20639877  20639989  20640748  20640937  20641083  19636371  21600095  21607290  21609651  21610023  21610258  21611144  21611240  21611337  21612941 | R  R  R  R  R  R  R  R  R  R  R  R  R  S  R  S | S  S  S  S  S  S  S  R  S  S  S  S  S  S  S  S | S  S  S  R  S  R  S  R  S  S  S  S  S  S  S  S | S  S  S  R  S  R  S  R  S  S  S  S  S  S  S  S | S  S  R  R  S  S  R  R  S  R  R  R  S  R  S  S | S  S  S  S  S  S  S  S  S  S  S  S  S  S  S  S | S  S  S  S  S  S  S  S  S  S  S  S  S  S  S  S | S  S  S  S  S  S  S  S  S  S  S  S  S  S  S  S | R  R  R  R  R  R  R  R  R  R  R  R  R  R  R  S | S  S  S  S  S  S  S  S  S  S  S  S  S  S  S  S | S  S  S  R  S  R  S  R  S  S  S  I  I  S  S  S | S  S  S  S  S  S  S  S  S  S  S  S  S  S  S  S | R  S  R  R  R  R  I  R  S  R  R  R  S  R  R  S | S  S  S  S  S  S  S  S  S  S  S  S  S  S  S  S | S  S  R  R  S  R  S  R  S  R  S  S  S  R  S  S |

Notes: penicillin G-G, gentamicin-CN, levofloxacin-LVX, Linezolid -LZD, Quinupristin/dalfopristin -QD, Tigecycline -TGC, Ciprofloxacin- CIP, clindamycin-CLI, oxacillin-OX, moxifloxacin-MXF, sulfamethoxazole compound-SMZ-TMP, erythromycin-E, Vancomycin -VA, Tetracycline-TE

^a-o^ The results present the minimum inhibitory concentration (MIC, μg/mL)

a Penicillin G (MIC, μg/mL): resistant (R) ≥0.25; susceptible (S) ≤0.125.

b Gentamicin (MIC, μg/mL): resistant (R) ≥16; intermediate (I) 4-16; susceptible (S) ≤4.

c Moxifloxacin (MIC, μg/mL): resistant (R) ≥2; intermediate (I) 0.5-2; susceptible (S) ≤0.5.

d Levofloxacin (MIC, μg/mL): resistant (R) ≥4; intermediate (I) 1-4; susceptible (S) ≤1.

e Clindamycin (MIC, μg/mL):resistant (R) ≥4; intermediate (I) 0.5-4; susceptible (S) ≤0.5.

f Linezolid(MIC, μg/mL):resistant (R) ≥4; intermediate (I) 0.5-4; susceptible (S) ≤0.5.

g Quinuptin /dalfopristin (MIC, μg/mL):resistant (R) ≥4; intermediate (I) 0.5-4; susceptible (S) ≤0.5.

h Tigecycline (MIC, μg/mL):resistant (R) ≥4; intermediate (I) 0.5-4; susceptible (S) ≤0.5.

i Oxacillin (MIC, μg/mL): resistant (R) ≥4; intermediate (I) 2-4; susceptible (S) ≤2.

j Ciprofloxacin (MIC, μg/mL): resistant (R) ≥4; intermediate (I) 1-4; susceptible (S) ≤1..

k Compound xinnuoming (MIC, μg/mL): resistant (R) ≥4; intermediate (I) 2-4; susceptible (S) ≤2.

l Erythrocin (MIC, μg/mL): resistant (R) ≥8; intermediate (I) 0.5-8; susceptible (S) ≤0.5.

m Erythromycin (MIC, μg/mL): resistant (R) ≥8; intermediate (I) 0.5-8; susceptible (S) ≤0.5.

n Vancomycin (MIC, μg/mL): resistant (R) ≥16; intermediate (I) 4-16; susceptible (S) ≤4.

o Tetracycline (MIC, μg/mL): resistant (R) ≥16; intermediate (I) 4-16; susceptible (S)≤4. **Supplementary Table** 4 Important differential expression genes between PG-treated *S. aureus* (USA 300_EG) and non-treated S. aureus (USA 300_CK) groups

| \| GeneID \| \| --- \| | log2Ratio(USA300_EG/USA300_CK) | Up-Down-Regulation  (USA300_EG/USA300_CK) | P-value |
| --- | --- | --- | --- | --- |
| glmU | -1.54815 | down | 5.37E-07 |
| murQ | -3.04921 | down | 5.06E-19 |
| nagA | -1.0429 | down | 0.000909 |
| aaa | -3.05752 | down | 2.82E-23 |
| vraR | 1.882037 | up | 2.50E-10 |
| dltD | 1.130767 | up | 0.000102 |
| dltB | 1.062283 | up | 0.000234 |
| sdrC | -4.62217 | down | 8.17E-14 |
| sdrD | -3.51742 | down | 2.61E-11 |
| ebpS | \| -0.99001 \|  \|  \| \| --- \| --- \| --- \| | down | 0.032934 |
| efb | -3.03287 | down | 6.41E-07 |
| sbi | -3.62143 | down | 3.26E-29 |
| isaA | -1.58953 | down | 2.42E-05 |
| isaB | -2.32995 | down | 2.87E-13 |
| ecb | -2.17855 | down | 1.77E-11 |
| scb | -2.28031 | down | 0.008131 |
| srtA | -2.57558 | down | 0.00106 |
| scdA | -4.19045 | down | 6.99E-05 |
| sdrE | 1.423293 | up | 8.98E-07 |
| sasA | 1.083377 | up | 0.000203 |
| arcc | -3.37715 | down | 1.11E-26 |
| argF | -4.8116 | down | 2.29E-43 |
| nreA | -7.95689 | down | 6.01E-33 |
| nreB | -3.81535 | down | 2.00E-16 |
| srrB | \| -1.1095 \|  \| 0.000129 \| \| --- \| --- \| --- \| | down | down |
| clpL | -3.63725 | down | 6.52E-31 |
| capN | 1.134564 | up | 0.0012 |
| cap8L | 1.166754 | up | 0.002263 |
| cap8F | 1.953216 | up | 4.32E-06 |
| cap8B | 3.621459 | up | 5.09E-17 |
| cap8G | 1.286025 | up | 0.001877 |
| cap8D | 2.690049 | up | 3.47E-12 |
| cap8C | 2.942556 | up | 2.50E-12 |
| cap8E | 1.16075 | up | 0.014166 |
| capA | 3.619638 | up | 2.82E-11 |
